# Oral administration of branched-chain amino acids ameliorates high-fat diet-induced metabolic-associated fatty liver disease *via* gut microbiota-associated mechanisms

**DOI:** 10.3389/fmicb.2022.920277

**Published:** 2022-07-22

**Authors:** Ranran Zhang, Hongna Mu, Ziyun Li, Jie Zeng, Qi Zhou, Hongxia Li, Siming Wang, Xianghui Li, Xianghui Zhao, Liang Sun, Wenxiang Chen, Jun Dong, Ruiyue Yang

**Affiliations:** ^1^The Key Laboratory of Geriatrics, Beijing Institute of Geriatrics, Institute of Geriatric Medicine, Chinese Academy of Medical Sciences, Beijing Hospital/National Center of Gerontology of National Health Commission, Beijing, China; ^2^Institute of Geriatrics, Peking University Fifth School of Clinical Medicine, Beijing, China; ^3^National Center for Clinical Laboratories, Institute of Geriatric Medicine, Chinese Academy of Medical Sciences, Beijing Hospital/National Center of Gerontology, Beijing, China

**Keywords:** branched-chain amino acids, metabolic-associated fatty liver disease, lipometabolism, gut microbiota dysbiosis, branched-chain amino acids metabolism, LC–MS/MS

## Abstract

Branched-chain amino acids (BCAAs), essential amino acids for the human body, are mainly obtained from food. High levels of BCAAs in circulation are considered as potential markers of metabolic-associated fatty liver disease (MAFLD) in humans. However, there are conflicting reports about the effects of supplement of BCAAs on MAFLD, and research on BCAAs and gut microbiota is not comprehensive. Here, C57BL/6J mice were fed with a high-fat diet with or without BCAAs to elucidate the effects of BCAAs on the gut microbiota and metabolic functions in a mouse model of MAFLD. Compared to high-fat diet (HFD) feeding, BCAA supplementation significantly reduced the mouse body weight, ratio of liver/body weight, hepatic lipid accumulation, serum levels of total cholesterol (TC), low-density lipoprotein cholesterol (LDL-C) and alanine aminotransferase (ALT), and the expressions of the lipogenesis-related enzymes Fas, Acc, and Scd-1 and increased expressions of the lipolysis-related enzymes Cpt1A and Atgl in the liver. BCAAs supplementation also counteracted HFD-induced elevations in serum BCAAs levels by stimulating the enzymatic activity of BCKDH. Furthermore, BCAAs supplementation markedly improved the gut bacterial diversity and altered the gut microbiota composition and abundances, especially those of genera, in association with MAFLD and BCAAs metabolism. These data suggest that BCAA treatment improves HFD-induced MAFLD through mechanisms involving intestinal microbes.

## Introduction

Metabolic-associated fatty liver disease (MAFLD) is characterized by excessive fat accumulation in the liver and is defined by lipid accumulation of more than 5% in hepatocytes as determined by histological analysis. The term MAFLD is derived from nonalcoholic fatty liver (NAFLD), which was defined by Ludwig in 1980 to describe the pathological syndrome characterized by bullous fatty change of more than 5%–10% of hepatocytes except for alcohol and other clear liver damaging factors (Ludwig et al., 1980). In 2020, the international consensus group proposed the new concept MAFLD, which can more accurately reflect the characteristics of fatty liver diseases related to metabolic dysfunction and should replace NAFLD. The prevalence of MAFLD makes it a common health concern worldwide, and research attention on the disease is gradually increasing. Although many factors, such as obesity, type 2 diabetes, and cardiovascular disease, have been implicated in MAFLD, the specific pathogenesis of MAFLD and effective treatment strategies remain elusive (Dietrich and Hellerbrand, 2014).

The pathological characteristic of MAFLD is excessive lipid accumulation, which results from an imbalance between lipid synthesis and lipid consumption. Hepatic lipid synthesis has been widely reported to be regulated by many key enzymes involved in the synthesis of fatty acids, including fatty acid synthase (Fas), acetyl-CoA carboxylase (Acc), and stearoyl-CoA desaturase (Scd1), of which expression levels are significantly increased in MAFLD model mice (Strable and Ntambi, 2010; Gao et al., 2020). On the other hand, lipolysis is the pathway responsible for the breakdown of triglycerides and the release of free fatty acids by adipose triglyceride lipase (Atgl), and free fatty acids are mostly consumed through fatty acid β-oxidation, which is limited by carnitine palmitoyltransferase 1A (Cpt1A; Li Q. et al., 2018). Increased expression of Atgl and Cpt1A decreases *de novo* lipogenesis in high-fat diet (HFD)-fed mice (Dusabimana et al., 2021). Together, the lipid accumulation in MAFLD is due to the disruption of lipid metabolism, denoting it a potential therapeutic target.

In the past 10 years, gut microbiota dysbiosis has been repeatedly observed in MAFLD, and gut microbiota markedly impacts lipid metabolism in the liver (Ma et al., 2017; Aron-Wisnewsky et al., 2020). Germ-free mice receiving microbiota from MAFLD model mice developed hepatic macrovesicular steatosis and exhibited increased liver concentrations of triglycerides and increased expression levels of Acc (Le Roy et al., 2013). In addition, gut-derived toxins, such as lipopolysaccharides (LPS), are reported to accelerate hepatic lipid accumulation and further influence the development of MAFLD by upregulating the expressions of Fas and Acc in mice livers (Endo et al., 2007; Chen et al., 2011). Therefore, understanding the correlation between microbiome characterization and MAFLD is critical for improving the treatment of MAFLD.

Branched-chain amino acids (BCAAs), including leucine, isoleucine, and valine, are essential amino acids that cannot be synthesized by metazoans and are therefore obtained most efficiently by diet. BCAAs play a critical role in the regulation of energy homeostasis, nutrition metabolism, gut health, immunity, and diseases in humans and animals (Nie et al., 2018). Several epidemiological studies indicate that elevated circulating level of BCAAs is a potential risk factor for MAFLD in humans (Flores-Guerrero et al., 2019; van den Berg et al., 2019). However, there are some conflicting reports on experimental animals. BCAAs supplementation in obese/diabetic mice appears to suppress hepatic *de novo* lipogenesis and promote adipocyte lipolysis, causing abnormal lipolysis and hyperlipidemia, and liver injury (Zhang et al., 2016; Iwao et al., 2020; Zhao et al., 2020). In contrast, research on nonalcoholic steatohepatitis mice showed that BCAAs supplementation alleviated hepatic steatosis and liver injury by suppressing the expression of lipogenesis-related genes and proteins (Honda et al., 2017). Thus, it is necessary to further clarify the effect of BCAAs on the liver and the possible mechanisms. Moreover, the gut is capable of regulating the biosynthesis, transport, and metabolism of BCAAs (Pedersen et al., 2016; Yoon, 2016). However, research on the effects of BCAAs supplementation on MAFLD model mice and the connection between gut microbiota alterations and BCAAs treatment in mice with MAFLD induced by a high-fat diet is lacking. The objective of this study was to determine the effects of BCAAs administration on MAFLD in HFD-fed mice and its underlying mechanisms, paying particular attention to its influence on gut microbiota remodeling and BCAAs metabolism.

## Materials and methods

### Animal studies

Six-week-old male C57BL/6J mice were purchased from Vital River Laboratory Animal Technology Co., Ltd. (Beijing, China) and raised (*n* = 3–4 mice/cage) in a special room with a temperature of 22°C and a 12 h light/dark cycle. The normal chow diet (ND, 12% of kcal fat, 1025), ND supplemented with BCAAs (ND + BCAAs), HFD [41% of kcal fat with an extra supplement of 0.15% (w/w) cholesterol, H10141], and HFD supplemented with BCAAs (HFD + BCAAs) were purchased from Beijing HFK Bioscience Co., Ltd. (Beijing, China). Valine, L-leucine, and L-isoleucine were purchased from Nanjing Jingzhu Biotechnology Co., Ltd. (Nanjing, China). The absolute amounts of L-leucine, L-isoleucine and valine per 100 g diet supplemented with BCAAs were 0.56, 0.40, and 0.40 g, respectively. All the mice had free access to food and water. Throughout the experiment, the padding and water were changed once a week, while the HFD and HFD + BCAAs food were changed twice a week to prevent sudor production *via* fat oxidation, thereby affecting the establishment of the model.

### Groups and treatments

After 1 week of acclimation to the ND diet, the mice were randomly divided into the following four groups and treated for 16 weeks: ND group (*n* = 10), ND + BCAAs group (*n* = 10), HFD group (*n* = 10), and HFD + BCAAs group (*n* = 10). Body weight was measured every 2 weeks. After 16 weeks of feeding, the mice were anesthetized by pentobarbital (30 mg/kg) and sacrificed after blood samples were collected by cardiac puncture. Their livers were collected, weighed, frozen in liquid nitrogen, and stored at −80°C until use. The ileal, cecal, and colonic contents were also aseptically collected, frozen in liquid nitrogen, and stored at −80°C until use. All these treatments were approved by the Peking University Biomedical Ethics Committee Experimental Animal Ethics Branch (LA2013-73) and complied with the Guide for the Care and Use of Laboratory Animals established by the National Institutes of Health (NIH).

### Histological analysis

Liver tissues were collected and fixed with 4% paraformaldehyde for staining immediately after sacrifice. Liver tissues were paraffin-embedded, dewaxed, and rehydrated. Finally, the paraffin sections (5 μm) were stained with HE. For oil red O staining, frozen sections (8 μm) were stained with oil red O according to standard protocols.

### Biochemical assays

Briefly, 7180 automatic biochemical analyzers (Hitachi Ltd., Tokyo, Japan) were used to detect biochemical parameters, including total cholesterol (TC), triglycerides (TG), high-density lipoprotein cholesterol (HDL-C), low-density lipoprotein cholesterol (LDL-C), glucose (Roche, Indianapolis, IN, United States), and alanine aminotransferase (ALT, BioSino Bio-Technology & Science Inc., Beijing, China), according to the manufacturer’s instructions.

### LC–MS/MS

The serum BCAAs levels (Val, Ile, and Leu) were measured using our previously reported isotope dilution liquid chromatography–tandem mass spectrometry (LC/MS/MS) method (Yang et al., 2013). Briefly, 0.05 ml aliquots of calibrators or mouse serum samples were mixed with 0.05 ml of the isotopically labeled internal standard. The mixture was injected directly for determination after deproteinization with acetonitrile containing 0.1% formic acid. The extracted amino acids were separated on a Kinetex HILIC silica column (2.6 μm, 2.1 mm × 150 mm). Then, these amino acids were detected with electrospray ionization (ESI) in positive ionization mode by using multiple reaction monitoring (MRM).

### Western blot analysis

Western blot analysis was used to detect protein expression. Total protein was extracted from mouse livers using RIPA lysis buffer (Applygen Technologies, Beijing, China), and protein concentrations were determined using a BCA protein assay kit (Applygen Technologies, Beijing, China). Thirty micrograms of total protein per lane were separated on 10% SDS–PAGE gels and transferred onto PVDF membranes (Roche, Indianapolis, IN, United States). The membranes were blocked with TBST containing 3% nonfat milk for 2 h at room temperature and then incubated with primary antibodies overnight at 4°C. The primary antibodies against Acc, β-actin, Fas, Scd1, BCKDH-E1α, p-BCKDH-E1α, and Atgl were purchased from Cell Signaling Technology (Beverly, MA, United States). The primary antibody against Cpt1A was purchased from Abcam (Cambridge, MA, United States). After binding with secondary antibodies for 2 h at room temperature, the bands were detected using an ECL detection kit (Applygen Technologies, Beijing, China). Band intensity was assessed by densitometry and expressed as the mean density area using ImageJ analysis software.

### Gut microbiota analysis

The mouse ileum, cecum, and colon contents were collected at the end of the experiments for 16S ribosomal RNA (rRNA) gene sequencing and stored at −80°C after being snap-frozen in liquid nitrogen. The primers 338F (5′-ACTCCTACGGGAGGCAGCA-3′) and 806R (5′-GGACTACHVGGGTWTCTAAT-3′) were used to amplify hypervariable region 3 (V3) and hypervariable region 4 (V4) of bacterial 16S rRNA for Illumina deep sequencing. PCR amplification was conducted using high-fidelity DNA polymerase according to the manufacturer’s protocol (TransGen Biotech, Beijing, China). The library construction and sequencing steps were performed by Beijing Biomarker Technologies Co., Ltd. All the results were based on sequenced reads and operational taxonomic units (OTUs).

### Statistical analysis

All data are expressed as the mean ± standard error of the mean (SEM). Statistical analysis was performed with one-way analysis of variance (ANOVA) followed by Tukey’s *post hoc* test using GraphPad Prism 8. Alpha diversity indices, including ACE and Chao1, and beta diversity indices, including principal coordinate analysis (PCoA) and nonmetric multidimensional scaling (NMDS), were assessed using QI-IME. The community structures of the samples were drawn using R language tools. The different genera were chosen for heatmaps when the q value ≤0.05 (obtained after value of *p* correction) for comparisons between the following groups: ND vs. ND + BCAAs, ND vs. HFD, and HFD vs. HFD + BCAAs. Correlation analysis was conducted for the different filtered fecal bacteria and MAFLD-related parameters using Spearman’s rank correlation. A *p* ≤ 0.05 was considered as statistically significant.

## Results

### BCAAs supplementation relieves BCAAs metabolic disorders induced by HFD

**Figure 1:**
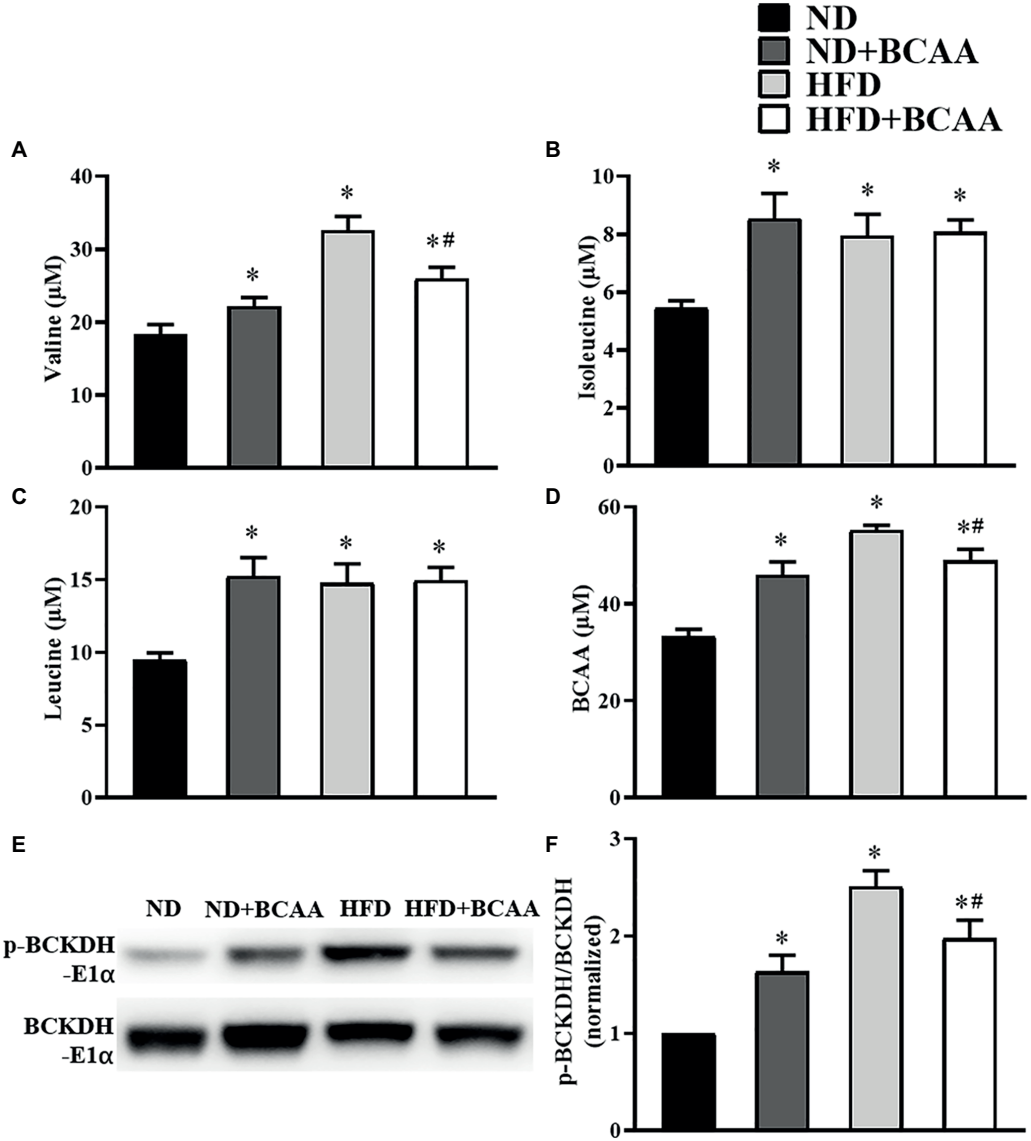
Effects of branched-chain amino acids (BCAAs) supplementation on BCAAs metabolic disorders induced by high-fat diet (HFD). Serum **(A)** valine, **(B)** isoleucine, **(C)** leucine, and **(D)** total BCAAs concentrations (*n* = 10 for the ND, ND + BCAAs and HFD groups, *n* = 8 for the HFD + BCAAs group). **(E)** The protein expression of p-BCKDH-E1α and BCKDH-E1α in the liver. **(F)** The relative quantitative data for p-BCKDH-E1α/BCKDH-E1α as indicated (*n* = 8). The densitometry is normalized to β-actin. The data are presented as the mean ± SEM. *p < 0.05 vs. the ND group; #*p* < 0.05 vs. the HFD group.

The serum concentrations of BCAAs were detected by LC–MS/MS, and the serum concentrations of valine, isoleucine, leucine, and total BCAAs were significantly higher in the HFD group than in the ND group. Moreover, the serum concentrations of valine and total BCAAs in the HFD + BCAAs group were significantly decreased compared to those in the HFD group. In addition, mice in the ND + BCAAs group showed significantly higher levels of valine, isoleucine, leucine, and total BCAAs than those in the ND group (Figures 1A–D). Western blot results showed that the ratio of p-BCKDH-E1α and BCKDH-E1α expression in the HFD group was significantly higher than that in the ND group, while the ratio in the HFD + BCAAs group was lower than that in the HFD group. Compared to that in the ND group, this ratio was increased in the ND + BCAAs group (Figures 1E,F). Together, these data demonstrate that BCAAs supplementation can relieve HFD-induced BCAAs catabolism disorders and serum BCAAs accumulation by stimulating the enzymatic activity of BCKDH.

### BCAAs supplementation inhibits the hepatic lipid accumulation in HFD-fed mice

**Figure 2:**
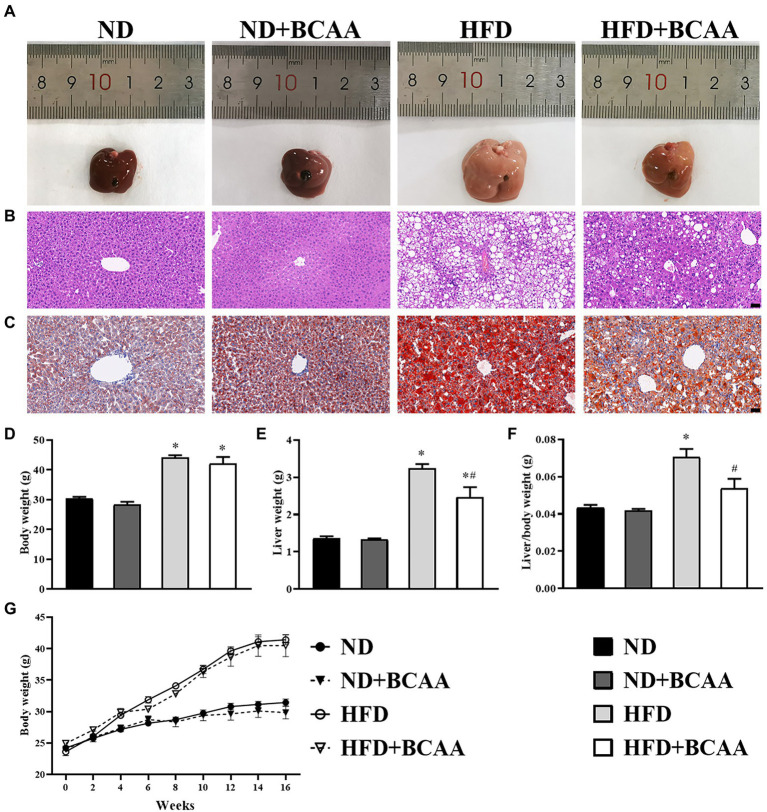
Effect of BCAAs supplementation on the hepatic lipid accumulation in mice. **(A)** Macroscopic view of livers from the different groups. Representative images of the light microscopic **(B)** HE and **(C)** oil red O staining of liver tissues from the different groups, bar = 50 μm. **(D,G)** Body weights (*n* = 9 for the ND, ND + BCAAs and HFD groups, *n* = 10 for the ND + BCAAs group), **(E)** liver weights (*n* = 4), and **(F)** liver/body weight ratios (*n* = 4). The data are presented as the mean ± SEM. *p < 0.05 vs. the ND group; #*p* < 0.05 vs. the HFD group.

To evaluate the effect of BCAAs supplementation on hepatic lipid accumulation, histological experiments were carried out. The liver tissues of ND mice were dark red, yellowish, and swollen. Notably, the abnormal appearance of livers induced by HFD feeding was relieved in HFD + BCAAs mice. Compared with those in the ND group, the livers of mice in the ND + BCAAs group were not significantly different (Figure 2A). As shown in Figure 2B, HE staining of liver histological slices showed neatly arranged hepatocytes in the ND group, while serious steatosis of liver cells and many large lipid vacuoles in the hepatocytes were observed in the HFD group. Compared with those in the HFD group, the steatosis and vacuolization in the HFD + BCAAs group were markedly alleviated (Figure 2B). Oil red O staining of liver histological slices also showed that lipid droplets were increased in the HFD group compared with the ND group and BCAAs-treated mice exhibited significantly less fat deposition, resulting in a reduction in MAFLD (Figure 2C). In addition, in our *in vivo* model of MAFLD, feeding of the high-fat diet increased the body weight, liver weight, and ratio of liver weight to body weight. Interestingly, BCAAs supplementation resulted in reduced liver weights and liver/body weight gains in HFD mice. Compared with those in the ND group, the morphology of livers, body weights, liver weights, and liver/body weights of mice in the ND + BCAAs group were not significantly different (Figures 2D–G). Thus, these findings suggest that BCAAs supplementation inhibits the hepatic lipid accumulation in HFD-fed mice.

### BCAAs supplementation improves the lipid metabolism in HFD-fed mice

**Figure 3:**
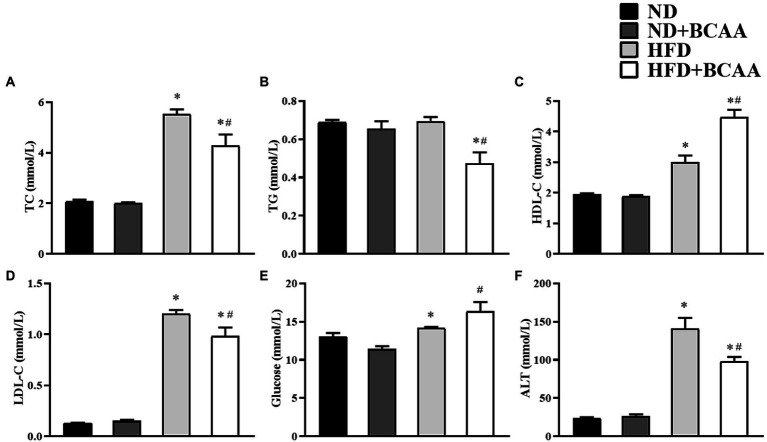
Effect of BCAAs supplementation on the blood lipid, glucose, and ALT levels in mice. The serum concentrations of **(A)** total cholesterol (TC), **(B)** triglycerides (TGs), **(C)** high-density lipoprotein cholesterol (HDL-C), **(D)** low-density lipoprotein cholesterol (LDL-C), **(E)** glucose, and **(F)** alanine aminotransferase (ALT). The data are presented as the mean ± SEM (*n* = 6). *p < 0.05 vs. the ND group; #*p* < 0.05 vs. the HFD group.

As shown in Figure 3, the HFD group exhibited significantly higher concentrations of TC, HDL-C, LDL-C, and ALT than the ND group. Mice in the HFD + BCAAs group had significantly decreased TC, TG, LDL-C, and ALT levels compared to those in the HFD group but increased glucose levels. No significant variations were observed between the ND + BCAAs and ND groups (Figure 3). These results suggest that BCAAs administration protects mice from lipid metabolism disorders and liver function abnormalities induced by HFD feeding.

### BCAAs supplementation inhibits the hepatic fatty acid synthesis in HFD-fed mice

**Figure 4:**
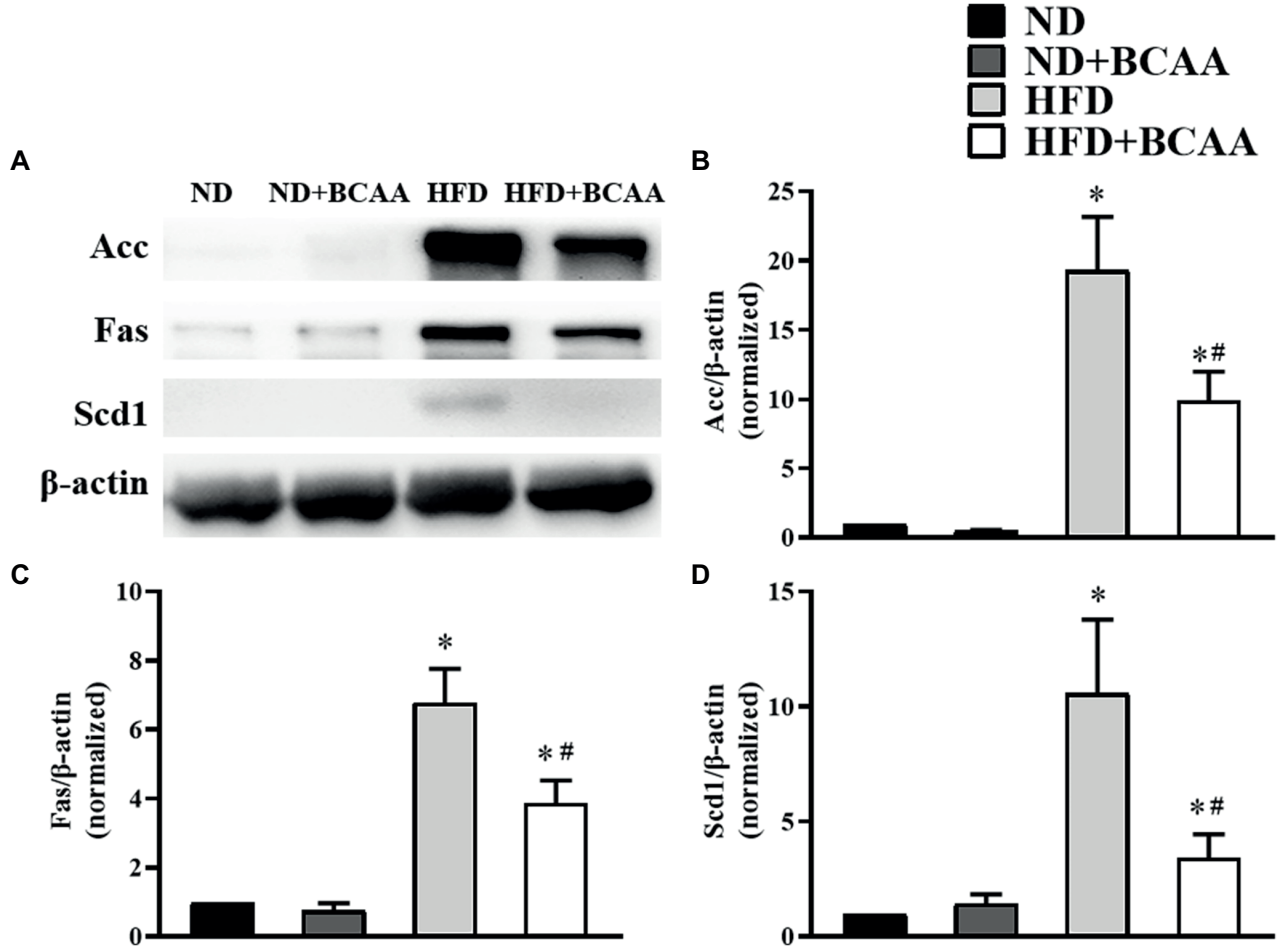
Effect of BCAAs supplementation on the hepatic fatty acid synthesis in HFD-fed mice. **(A)** The protein expression of acetyl-CoA carboxylase (Acc), fatty acid synthase (Fas), and stearoyl-CoA desaturase 1 (Scd1) in the liver. The relative quantitative data for **(B)** Acc, **(C)** Fas, and **(D)** Scd1 as indicated. The densitometries were normalized to β-actin. The data are presented as the mean ± SEM (*n* = 6). *p < 0.05 vs. the ND group; #*p* < 0.05 vs. the HFD group.

Given their roles in fatty acid biosynthesis, Acc, Fas, and Scd1 are key factors in the pathogenesis of hepatic fatty acid accumulation. In our model of MAFLD, the HFD-fed mice showed significantly increased protein expression levels of Acc, Fas, and Scd1 compared to those in the ND mice. However, BCAAs supplementation decreased the expression levels of Acc, Fas, and Scd1 in the HFD-fed mice. No significant variations were observed between the ND + BCAAs and ND groups (Figure 4). Together, these results indicate that the suppression of hepatic fatty acid synthesis may be a mechanism by which BCAAs supplementation inhibits lipid accumulation.

### BCAAs supplementation promotes hepatic lipid hydrolysis and oxidation in HFD-fed mice

**Figure 5:**
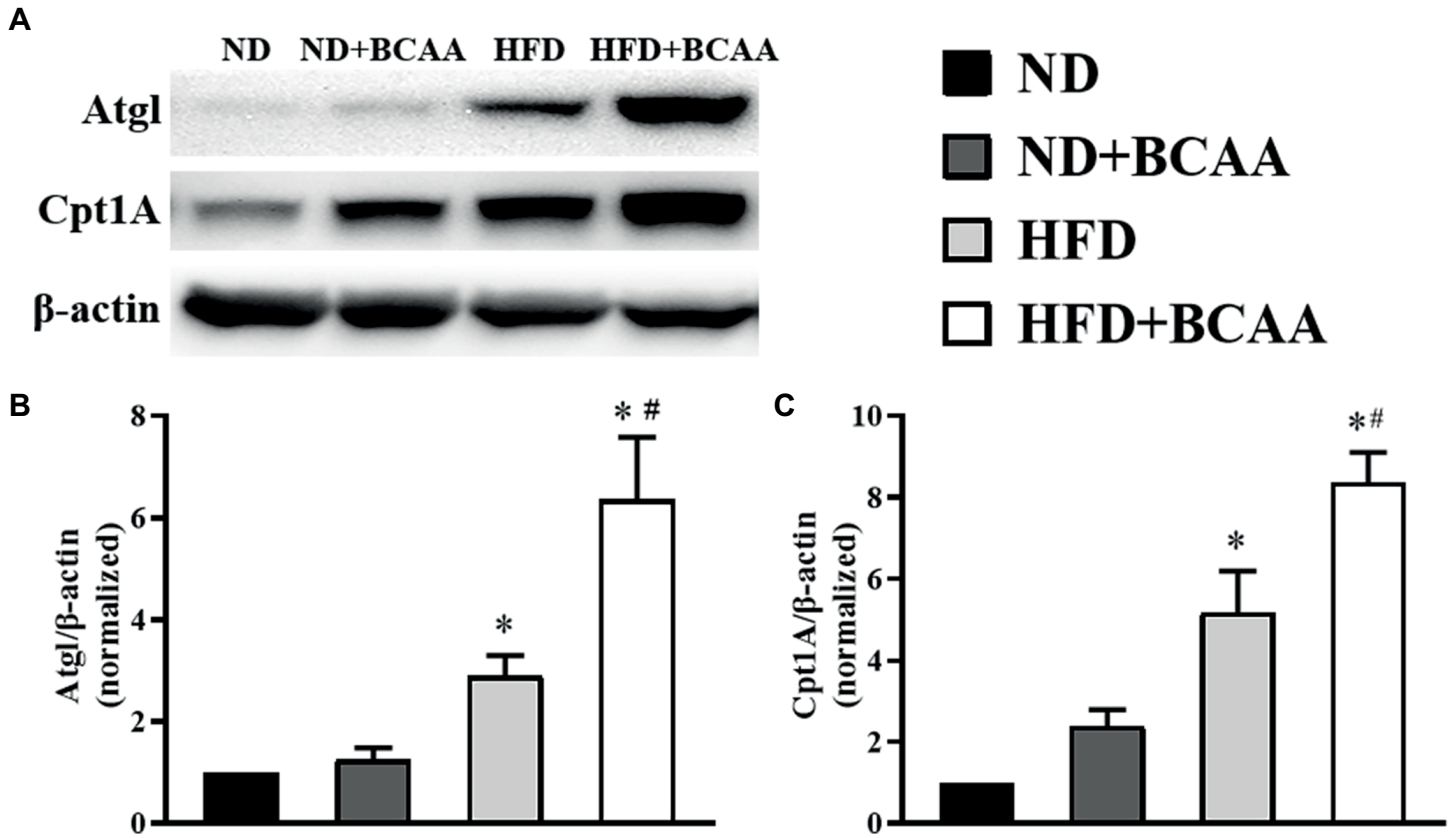
Effect of BCAAs on the lipid hydrolysis and oxidation-related protein expression in HFD-fed mice. The protein expression of **(A)** adipocyte triglyceride lipase (Atgl) and carnitine palmitoyl transferase 1A (Cpt1A) in the liver. The relative quantitative data for **(B)** Atgl and **(C)** Cpt1A as indicated. The densitometries were normalized to β-actin (*n* = 6). The data are presented as the mean ± SEM. *p < 0.05 vs. the ND group; #*p* < 0.05 vs. the HFD group.

We next assessed the effect of BCAAs administration on the expression of proteins involved in hydrolysis and fatty acid oxidation in the liver by western blot. Atgl catalyzes the hydrolysis of the first ester bond of lipid molecules, and Cpt1A catalyzes the rate-limiting step of fatty acid β-oxidation. Both of these enzymes are necessary for fatty acid decomposition. As shown in Figure 5, the expression levels of Atgl and Cpt1A were higher in the HFD group than in the ND group, and they were further accelerated by BCAAs supplementation in both the ND + BCAAs group and the HFD + BCAAs group. These results indicate that promoting lipid hydrolysis and oxidation may be another mechanism by which BCAAs inhibit hepatic lipid accumulation.

### BCAAs supplementation improves the bacterial diversity in the gut

**Figure 6:**
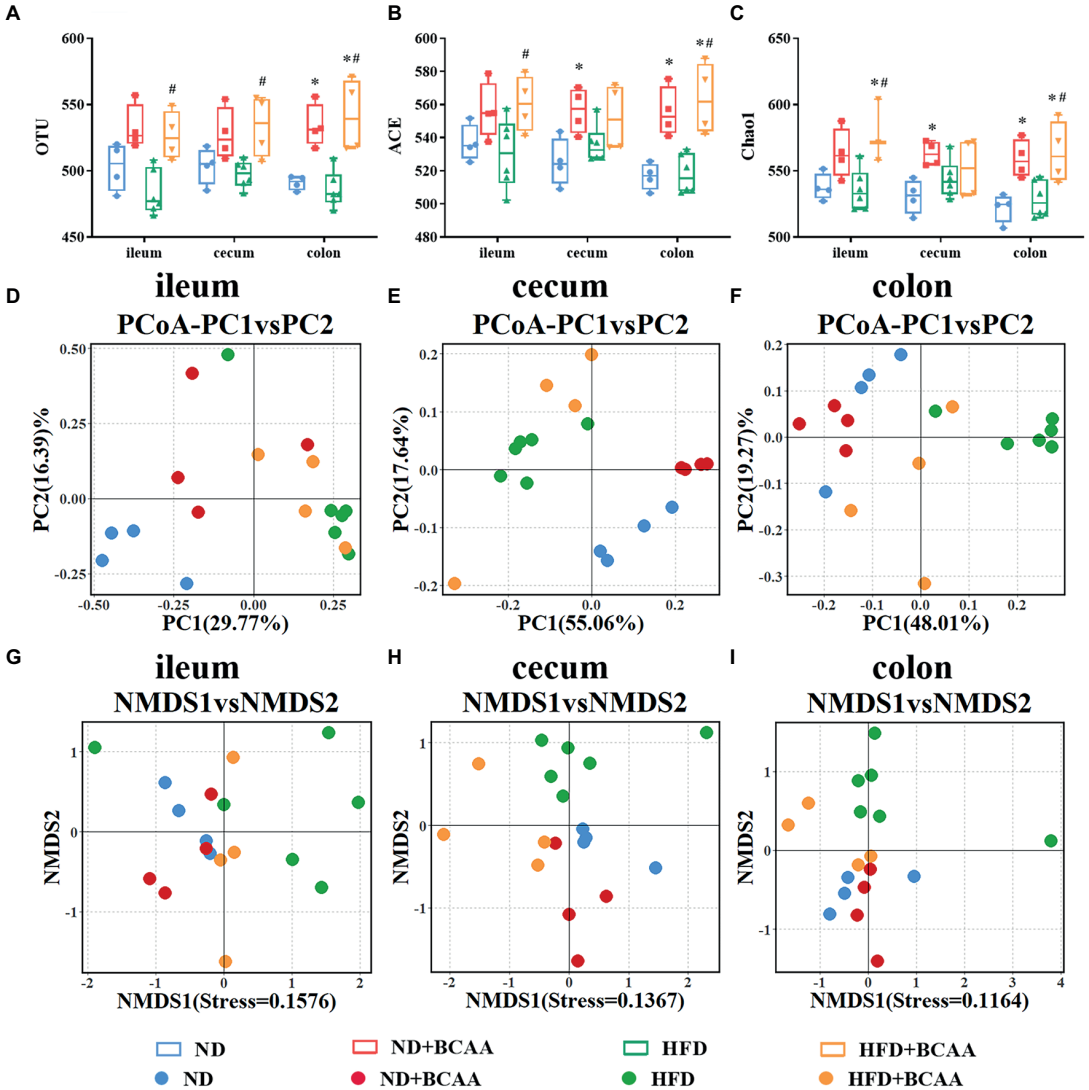
Effect of BCAAs supplementation on gut bacterial diversity. **(A)** operational taxonomic unit (OTU), **(B)** ACE, and **(C)** Chao1 analyses of the gut bacteria from different parts of the gut in the indicated groups. **(D–F)** Principal coordinate analysis (PCoA) analysis of the microbiota composition. **(G–I)** Nonmetric multidimensional scaling (NMDS) analysis of the microbiota composition (*n* = 4 for the ND, ND + BCAAs, and HFD + BCAAs groups, *n* = 6 for the HFD group). *p < 0.05 vs. the ND group; #*p* < 0.05 vs. the HFD group.

To determine the effect of BCAAs supplementation on gut microbial diversity, we investigated the compositions of gut microbiota in different groups by using 16S rRNA gene sequencing. Generally, microbial diversity can be quantified utilizing two metrics: alpha diversity and beta diversity. Alpha diversity analysis revealed that the gut microbial diversity was significantly increased in the HFD + BCAAs group compared with the HFD group. The observed OTUs of the gut microbiota from the ileum, cecum, and colon were obviously increased in the HFD + BCAAs group compared with the HFD group. Increasing trends were also observed in the OTUs of the ileum, cecum, and colon in ND + BCAAs mice compared with ND mice. However, the OTUs were not obviously different between the ND and HFD groups (Figure 6A). In addition, ACE (Figure 6B), and Chao1 (Figure 6C) analyses were conducted. The changes in the ACE and Chao1 were similar to the OTU results. These results suggest that BCAAs supplementation improves the richness and diversity of the gut microbiota in mice.

Beta diversity analysis was employed to assess differences in the global bacterial compositions among the four groups. The results of PCoA based on the Bray-Curtis distance and NMDS based on the Jaccard distance are presented in Figures 6D–I, respectively. PCoA and NMDS can be used to show the distinct and separate clustering of microbiota compositions among all treatment groups. These analyses demonstrate that BCAAs supplementation altered the composition and improved the relative abundances of gut microbiota, especially in the cecum and colon.

**Figure 7:**
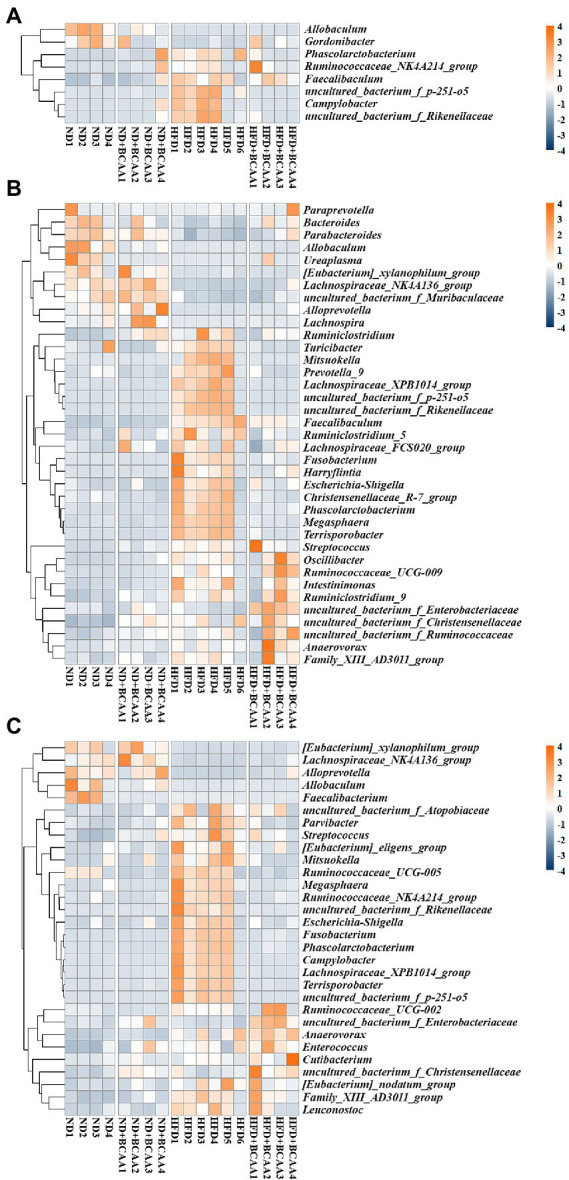
Effect of BCAAs supplementation on the gut microbiota composition. Hierarchically clustered heatmaps of gut microbiota that were significantly altered (ND vs. ND + BCAAs, ND vs. HFD, HFD vs. HFD + BCAAs, *q* ≤ 0.05 after correcting for the value of *p*) in the **(A)** ileum, **(B)** cecum, and **(C)** colon at the genus level. The scale bar shows the standardized *Z*-value of the microbial relative abundance. Red and blue colors indicate higher and lower mean expression, respectively (*n* = 4 for the ND, ND + BCAAs, and HFD + BCAAs groups, *n* = 6 for the HFD group of biologically independent mice).

**Figure 8:**
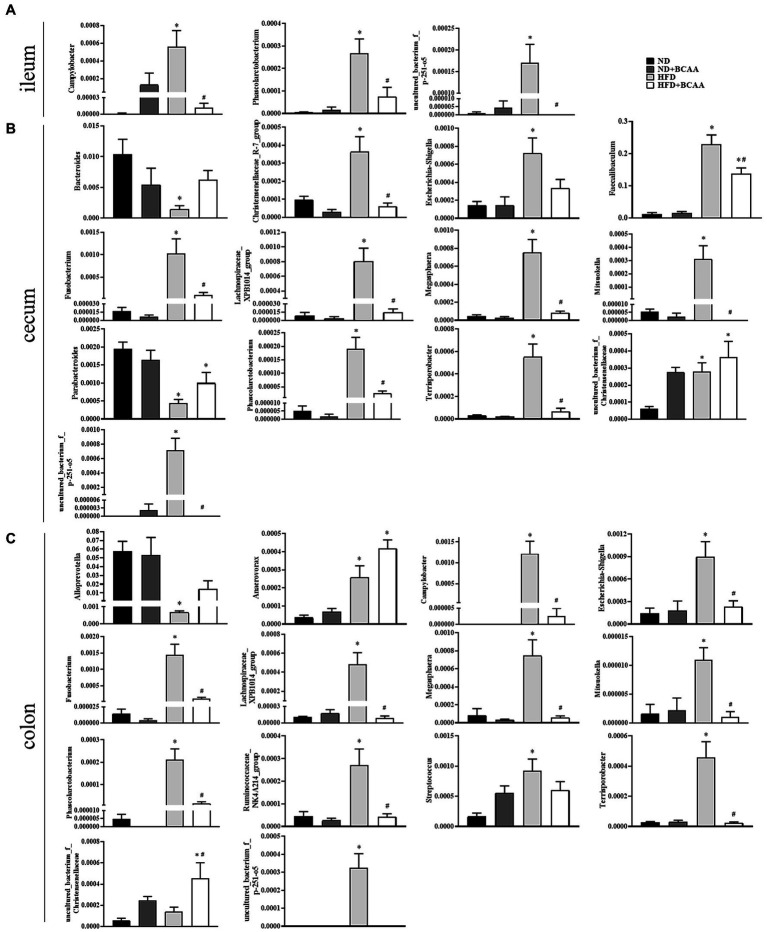
Effect of BCAAs supplementation on the relative abundances of gut bacteria. **(A)** Relative abundances of *Campylobacter*, *Phascolarctobacterium*, and *uncultured_bacterium_f_p-251-o5* in the ileum. **(B)** Relative abundances of *Bacteroides*, *Christensenellaceae_R-7_group*, *Escherichia-Shigella*, *Faecalibaculum*, *Fusobacterium*, *Lachnospiraceae_XPB1014_group*, *Megasphaera*, *Mitsuokella*, *Parabacteroides*, *Phascolarctobacterium*, *Terrisporobacter*, *uncultured_bacterium_f_Christensenellaceae*, and *uncultured_bacterium_f_p-251-o5* in the cecum. **(C)** Relative abundances of *Alloprevotella*, *Anaerovorax*, *Campylobacter*, *Escherichia-Shigella*, *Fusobacterium*, *Lachnospiraceae_XPB1014_group*, *Megasphaera*, *Mitsuokella*, *Phascolarctobacterium*, *Ruminococcaceae_NK4A214_group*, *Streptococcus*, *Terrisporobacter*, *uncultured_bacterium_f_Christensenellaceae*, and *uncultured_bacterium_f_p-251-o5* in the colon. *p < 0.05 vs. the ND group; #*p* < 0.05 vs. the HFD group.

Heatmaps of different (ND vs. ND + BCAAs, ND vs. HFD, HFD vs. HFD + BCAAs, q ≤ 0.05 after correcting for the value of *p*) genera strongly revealed that BCAAs supplementation reshaped the HFD-induced changes in the gut bacteria profile (Figures 7A–C). Compared with those in the ND group, six genera in the ileum, 17 genera in the cecum, and 11 genera in the colon all presented relatively high abundances in the HFD group. In addition, the relative abundances of all these genera were decreased in the HFD + BCAAs group. In contrast, one genus in the ileum and two genera in the cecum had relatively low abundances compared with those in the HFD group, while these differences were incremental in the HFD + BCAAs group. In addition, eight genera in the cecum and five genera in the colon all had relatively higher abundances in the HFD groups than in the ND group, and these were further augmented in the HFD + BCAAs group. Figure 8 shows the statistical results for the bacteria with significantly altered abundances. Collectively, these results further support the modifying effect of BCAAs supplementation on the gut microbiota.

### Correlation between the gut microbiota and mafld-related parameters

**Figure 9:**
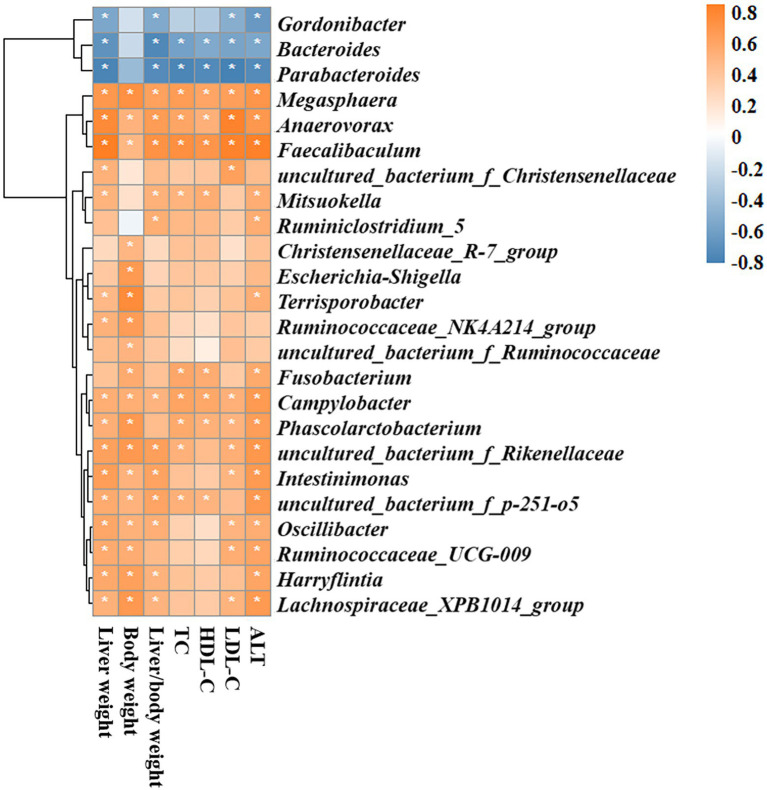
Spearman rank correlation analyses of the gut microbiota and MAFLD-related parameters. Red and blue colors indicate positive and negative correlation coefficients, respectively. Significant correlations are indicated by *p < 0.05.

Because BCAAs altered the MAFLD-related parameters and the gut bacterial composition in HFD-fed mice as mentioned above, we analyzed the correlations between gut microbiota and MAFLD-related parameters, including liver weight, body weight, liver/body weight ratio, TC, HDL-C, LDL-C, and ALT. *Gordonibacter*, *Bacteroides*, and *Parabacteroides* were significantly and negatively correlated with most MAFLD parameters. In contrast, *Megasphaera*, *Faecalibaculum*, *Mitsuokella*, *Fusobacterium*, *Campylobacter*, *Phascolarctobacterium*, *uncultured_bacterium_f_Rikenekkaceae*, *uncultured_bacterium_f_p-251-o5*, *Harryflintia*, and *Lachnospiraceae_XPB1014_group* were significantly and positively associated with MAFLD parameters. Other genera, including *uncultured_bacterium_f_Christensenellaceae*, *Ruminiclostridium_5*, *Christensenellaceae_R-7_group*, *Escherichia-Shigella*, *Terrisporobacter*, *Ruminococcaceae_NK4A214_group*, and *uncultured_bacterium_f_Ruminococcaceae* had positive correlations with several MAFLD parameters (Figure 9).

## Discussion

In the present study, we investigated the effect of BCAAs supplementation on lipid accumulation associated with MAFLD and its mechanism in HFD-fed mice. MAFLD was characterized by abundant lipid accumulation in the liver. BCAAs supplementation substantially relieved the lipid accumulation and BCAAs metabolic disorders induced by the HFD. BCAAs administration also ameliorated the levels of biochemical parameters, including TC, LDL-C, and ALT, and accelerated the degeneration of fatty acids by reducing expressions of the lipogenesis-related enzymes and increasing expressions of the lipolysis-related enzymes in the liver. In addition, BCAAs supplementation markedly improved the gut bacterial diversity and altered the gut microbiota composition and abundances, especially those of genera, in association with MAFLD and BCAAs metabolism. These results demonstrated that BCAAs supplementation can attenuate high-fat diet-induced MAFLD *via* gut microbiota-associated mechanisms.

Six-week-old male C57Bl/6 mice fed with a high-fat diet for 16 weeks have been established and subsequently confirmed as a typical MAFLD model (Li D. J. et al., 2018). This study demonstrated that an HFD led to BCAAs metabolic disorders, consistent with patients with MAFLD showing high serum levels of BCAAs, whereas BCAAs administration repaired the BCAAs metabolic blockade. BCAAs catabolism is regulated by branched-chain amino acid transaminase (BCAT) and BCKDH, which are particularly active in muscle and liver tissues (Iwasa et al., 2015; Holecek, 2018). BCKDH activity mainly depends on BCKDH-E1α, regulated by a phosphorylation-dephosphorylation mechanism. Phosphorylation of BCKDH-E1α mediated by BCKD kinase results in the inactivation of BCKDH, leading to elevated serum BCAAs concentrations (Burrage et al., 2014; Zhou et al., 2019). As the enzymatic activity of BCKDH-E1α was mainly observed in the liver, we detected its hepatic expression. As expected, the enzymatic activity of BCKDH was impaired by HFD feeding but stimulated by BCAAs supplementation with HFD feeding. Thus, dietary supplementation with BCAAs may lead to a boost in the BCAAs catabolic flux and counteract HFD-induced BCAAs catabolic injuries and the accumulation of BCAAs in circulation. However, it is worth noting that the inhibition of the enzymatic activity of BCKDH-E1α and elevated serum BCAAs levels were also observed in the normal diet supplemented with BCAAs. Indeed, the catabolic activity of BCAAs is susceptible to the influence of the nutrient environment (Sun and Wang, 2016). It has been reported that nutritional status regulates BCKD subunits and BCKDK expression in rats (Zhou et al., 2012). The homeostasis of BCAAs levels in circulation depends on the balance between BCAAs intake and catabolism, so we speculate that under different nutritional states caused by normal diet and high-fat diet, dietary supplementation with BCAAs may lead to different BCAAs metabolism.

MAFLD is characterized by abundant lipid accumulation in the liver. In this study, BCAAs were found to inhibit lipid accumulation in the liver. The alleviation of lipid accumulation in hepatocytes was more clearly shown by morphological, HE staining, and oil red O staining of liver tissues. Moreover, oral supplementation with BCAAs in HFD-fed mice counteracted the increase of liver weight by reducing liver steatosis due to reduced serum TC, TG, and LDL-C concentrations, supporting the protective effect of BCAAs on metabolic biomarkers of MAFLD. However, a previous study reported that the plasma TG and ALT concentrations in 8-week-old mice were increased after HFD supplementation with BCAAs for 12 weeks (Zhang et al., 2016). The discrepancies of the results are likely due to the different ratios and doses as well as the feeding period of the BCAAs. Our study adopted the recommended ratio and dose for humans, which is close to the daily intake range of BCAAs from a nutritional perspective.

An imbalance between lipid synthesis and lipid consumption is another pathological characteristic of MAFLD. The enzymes involved in fatty acid synthesis, such as Acc, Fas, and Scd1, play active roles in the production of excess fatty acids. Atgl initiates the hydrolysis of triglycerides to release fatty acids, and Cpt1A is an isomer that catalyzes the rate-limiting step of fatty acid oxidation in the liver (Schreiber et al., 2019; Schlaepfer and Joshi, 2020). To investigate the regulatory effect of BCAAs supplementation on hepatic lipid metabolism, we detected the expression of several key transcriptional regulators in the liver. In our study, the expression of Acc, Fas, and Scd1 was significantly increased in HFD-fed mice, and BCAAs supplementation inhibited the *de novo* fatty acid synthesis in the liver by downregulating these enzymes. Furthermore, the expression level of Cpt1A was increased in HFD-fed mice, which has been reported previously (Softic et al., 2019). At the same time, the expression of Atgl was increased in HFD-fed mice, and we speculate that the increasing trend in Atgl was probably due to the higher energy expenditure rather than the caloric intake in HFD-fed mice (Schreiber et al., 2019). BCAAs supplementation with an HFD further improved the expression levels of Atgl and Cpt1A. These results provide evidence that BCAAs administration disturbs the lipid accumulation in the liver by suppressing fatty acid synthesis and accelerating fatty acid catabolism, consistent with the morphological, histological, and biochemical parameters in the liver-related to MAFLD.

Increasing evidence supports the causative role of the gut microbiota in MAFLD development and progression (Tokuhara, 2021). MAFLD has been reported to be associated with gut microbiota dysbiosis, resulting in changes in the intestinal permeability, intestinal and systemic inflammatory responses, gut microbiota composition and metabolome, and bile acid profiles (Aron-Wisnewsky et al., 2013; DeJong et al., 2020). As expected, our study of the MAFLD model revealed differences at the genus level between HFD-fed mice and mice subjected to the other treatments. Alpha diversity analyses revealed that the richness and diversity of the gut bacteria were slightly decreased in HFD-fed mice and significantly reversed by BCAAs. Besides, the addition of BCAA to a normal diet may also change the diversity of gut microbiota. A previous study found the similar results and demonstrated that BCAA supplementation slowed down the change of gut microbiota caused by aging and reduces the serum concentration of lipopolysaccharide-binding protein in mice (Yang et al., 2016). Beta diversity analyses, including PCoA and NAMDs, indicated separate clusters of microbiotas between the ND group and the other treatment groups. These data implied that changes in gut bacteria might be partially responsible for the effect of BCAAs on the lipid accumulation in MAFLD model mice. In the current study, BCAAs supplementation in HFD-fed mice substantially affected the gut microbiota composition, counteracting gut microbiota dysbiosis.

To further identify the gut bacterial communities that differed from those in the ND mice, we analyzed the bacteria at the genus level. The relative abundances of genera such as *Gordonibacter*, *Bacteroides*, and *Parabacteroides* were negatively correlated with MAFLD-related parameters and were lower in the HFD group than in the ND group, while BCAAs supplementation in HFD-fed mice increased their relative abundances. Similarly, BCAAs-fed HFD increased the relative abundances of genera, such as *Megasphaera*, *Anaerovorax*, *Faecalibaculum*, *Campylobacter*, *Phascolarctobacterium*, *uncultured_bacterium_f_Rikenekkaceae*, *uncultured_bacterium_f_p-251-o5*, *Harryflintia*, and *Lachnospiraceae_XPB1014_group*. These genera, along with *uncultured_bacterium_f_Christensenellaceae*, *Ruminiclostridium_5*, *Christensenellaceae_R-7_group*, *Escherichia-Shigella*, *Terrisporobacter*, *Ruminococcaceae_NK4A214_group*, and *uncultured_bacterium_f_Ruminococcaceae* were positively associated with MAFLD parameters. These altered bacteria may participate in MAFLD progression, and BCAAs intervention leads to structural modulation of the gut bacteria, which might help mitigate MAFLD.

Gut microbiota play an important role in modifying host lipid metabolism (Schoeler and Caesar, 2019). Gut microbiota can produce short-chain fatty acids while carrying out their life activities. Short-chain fatty acids can inhibit liver fat synthase and redistribute cholesterol in blood and liver, thus reducing the level of blood lipids in serum (Jennison and Byrne, 2021). The transformation of cholesterol into bile acids is the main pathway of cholesterol metabolism. In general, the regulating effect of gut microbiota on blood lipids is mainly realized through its effect on cholesterol metabolism and mainly by promoting bile acid synthesis (Ebrahimzadeh Leylabadlo et al., 2020). In addition, when the intestinal environment changes, the growth of normal flora will be inhibited, and harmful flora will increase, resulting in intestinal flora imbalance. This imbalance can lead to abnormal blood lipid metabolism. In turn, abnormal blood lipid metabolism will further aggravate the imbalance of intestinal flora, forming a vicious circle. In our study, within these microbiotas, *Alloprevotella*, *Anaerovorax*, and *Bacteroides*, which have been reported to be related to short-chain fatty acid synthesis, were significantly inhibited by the high-fat diet, whereas the relative abundances of *Alloprevotella* and *Bacteroides* were reversed by BCAAs (Kong et al., 2019; Liu et al., 2020). As the leading cause of human bacterial gastroenteritis, the relative abundance of *Campylobacter* was efficiently increased by HFD feeding but inhibited by BCAAs administration in HFD-fed mice (Kaakoush et al., 2015). The abundances of *Escherichia-Shigella*, *Faecalibaculum*, and *Fusobacterium*, opportunistic pathogenic bacteria that may promote the inflammatory response and thus impair the intestinal barrier, were decreased in mice fed with a high-fat diet supplemented with BCAAs (Yin et al., 2013). The relative abundances of *Terrisporobacter*, which have been negatively associated with anti-inflammatory cytokine secretion, were lower in the HFD + BCAAs group than in the HFD group (Ho et al., 2019). Moreover, BCAAs supplementation in HFD mice increased the levels of *Parabacteroides*, which is capable of improving lipid metabolism disorders by hydrolyzing a variety of conjugated bile acids and converting them into secondary bile acids (Wang et al., 2019). A high-fat diet is more likely to increase the abundance of *Phascolarctobacterium*, which was observed in obese children, and the relative abundance of *Phascolarctobacterium* was increased in HFD mice but decreased in mice fed with an HFD and supplemented with BCAAs (Falalyeyeva et al., 2022).

More interestingly, the inhibitory effect of BCAAs administration on hepatic fat accumulation in HFD mice may have been due to the regulation of gut microbiota-mediated BCAAs metabolism. Elevated relative abundances of *Bacteroides*, which participate in BCAAs catabolism in the gut, and a decreased relative abundance of *Streptococcus*, which is involved in BCAAs biosynthesis, were observed in the HFD-fed mice supplemented with BCAAs, in accordance with the ameliorating effect of BCAAs supplementation on HFD-induced BCAAs catabolism disorders and serum BCAAs accumulation (Garault et al., 2000; Nyangale et al., 2012; Kim et al., 2017; Zeng et al., 2020). Combined with the circulating levels of BCAAs, our findings indicated that dietary supplementation with BCAAs may participate in MAFLD by altering the intestinal environment and BCAAs metabolism in the gut. Taken together, the gut microbiota plays a critical role in BCAAs metabolism, and BCAAs supplementation might have beneficial effects on hepatic steatosis by targeting gut microbiota related to MAFLD. However, more experiments should be conducted to elucidate the direct effect of BCAAs on the gut microbiota and specific signaling pathways between BCAAs and MAFLD. Further experiments need to be conducted to elucidate the direct effect of BCAAs on the gut microbiota and specific signaling pathways between BCAAs and MAFLD.

## Conclusion

**Figure 10:**
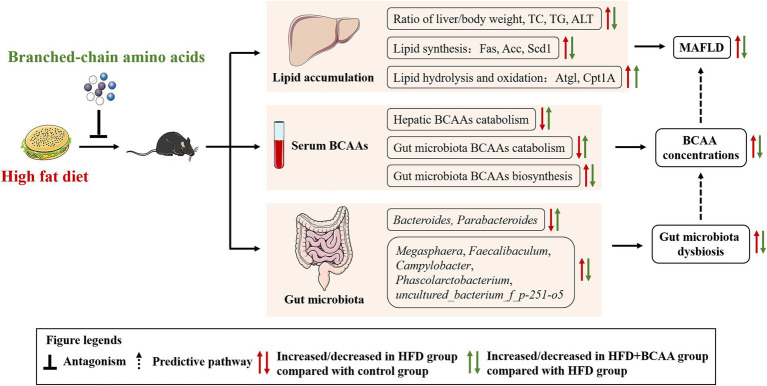
Proposed mechanisms for protective effects of BCAAs on HFD-fed mice.

We provide evidence that supplementation with BCAAs not only reduced lipid accumulation by inhibiting the expression of Acc, Fas, and Scd-1 and accelerating the expression of Atgl and Cpt1A but also altered the gut microbiota composition, which is related to MAFLD and BCAAs metabolism. Besides, we found that a high-fat diet increased the serum BCAAs levels by damaging BCAA catabolism, which was consistent with epidemiological statistics, while HFD supplementation with BCAAs was effective at attenuating BCAAs catabolism disorders and decreasing serum BCAAs concentrations. Our findings highlight the potential correlation between MAFLD and BCAAs metabolism, and BCAAs supplementation may improve hepatic steatosis and ameliorate HFD-induced fatty liver disease *via* gut microbiota-associated mechanisms (Figure 10).

## Data availability statement

The data presented in the study are deposited in NCBI Trace Archive NCBI Sequence Read Archive, bioProject number is PRJNA852193.

## Ethics statement

The animal study was reviewed and approved by the Peking University Biomedical Ethics Committee Experimental Animal Ethics Branch.

## Author contributions

RZ, HM, and RY: conceptualization. RZ and ZL: data curation and formal analysis. JD and RY: funding acquisition. RZ, ZL, JZ, HL, SW, XL, and XZ: investigation. HM, ZL, JZ, QZ, SW, XL, and LS: methodology. RZ and RY: project administration. XL and XZ: resources. HM, LS, WC, JD, and RY: supervision. RZ and QZ: visualization. RZ: writing—original draft. HM, JD, and RY: writing—review and editing. All authors contributed to the article and approved the submitted version.

## Funding

This work was supported by the National Natural Science Foundation of China (grant numbers 81672075 and 91849132), the Beijing Natural Science Foundation (grant numbers 7222156 and 7214250), the CAMS Innovation Fund for Medical Sciences (grant number 2021-I2M-1-050), the National Key R&D Program of China (grant number 2021YFE0114200), and the Priority Union Foundation of Yunnan Provincial Science and Technology Department (202001AY070001-011).

## Conflict of interest

The authors declare that the research was conducted in the absence of any commercial or financial relationships that could be construed as a potential conflict of interest.

## Publisher’s note

All claims expressed in this article are solely those of the authors and do not necessarily represent those of their affiliated organizations, or those of the publisher, the editors and the reviewers. Any product that may be evaluated in this article, or claim that may be made by its manufacturer, is not guaranteed or endorsed by the publisher.

## Abbreviations

Acc: Acetyl-CoA carboxylase
ALT: Alanine aminotransferase
Atgl: Adipocyte triglyceride lipase
BCAAs: Branched-chain amino acid
BCKDH: Branched-chain ketoacid dehydrogenase
BCKDH-E1α: Branched-chain α-keto acid decarboxylase
Cpt1A: Carnitine palmitoyl transferase 1A
Fas: Fatty acid synthase
HDL-C: High-density lipoprotein cholesterol
HFD: High-fat diet
LDL-C: Low-density lipoprotein cholesterol
MAFLD: Metabolic associated fatty liver disease
ND: Normal diet
Scd1: Stearoyl-CoA desaturase 1
TC: Total cholesterol
TG: Triglyceride

## References

[ref1] Aron-Wisnewsky J. Gaborit B. Dutour A. Clement K. (2013). Gut microbiota and non-alcoholic fatty liver disease: new insights. Clin. Microbiol. Infect. 19, 338–348. doi: 10.1111/1469-0691.12140, PMID: 23452163

[ref2] Aron-Wisnewsky J. Vigliotti C. Witjes J. Le P. Holleboom A. G. Verheij J.. (2020). Gut microbiota and human NAFLD: disentangling microbial signatures from metabolic disorders. Nat. Rev. Gastroenterol. Hepatol. 17, 279–297. doi: 10.1038/s41575-020-0269-9, PMID: 32152478

[ref3] Burrage L. C. Nagamani S. C. Campeau P. M. Lee B. H. (2014). Branched-chain amino acid metabolism: from rare Mendelian diseases to more common disorders. Hum. Mol. Genet. 23, R1–R8. doi: 10.1093/hmg/ddu123, PMID: 24651065 PMC4170715

[ref4] Chen X. Zhang C. Zhao M. Shi C. E. Zhu R. M. Wang H.. (2011). Melatonin alleviates lipopolysaccharide-induced hepatic SREBP-1c activation and lipid accumulation in mice. J. Pineal Res. 51, 416–425. doi: 10.1111/j.1600-079X.2011.00905.x, PMID: 21689150

[ref5] DeJong E. N. Surette M. G. Bowdish D. M. E. (2020). The gut microbiota and unhealthy aging: disentangling cause from consequence. Cell Host Microbe 28, 180–189. doi: 10.1016/j.chom.2020.07.013, PMID: 32791111

[ref6] Dietrich P. Hellerbrand C. (2014). Non-alcoholic fatty liver disease, obesity and the metabolic syndrome. Best Pract. Res. Clin. Gastroenterol. 28, 637–653. doi: 10.1016/j.bpg.2014.07.008 25194181

[ref7] Dusabimana T. Park E. J. Je J. Jeong K. Yun S. P. Kim H. J.. (2021). P2Y2R deficiency ameliorates hepatic steatosis by reducing lipogenesis and enhancing fatty acid beta-oxidation through AMPK and PGC-1alpha induction in high-fat diet-fed mice. Int. J. Mol. Sci. 22:5528. doi: 10.3390/ijms22115528, PMID: 34073834 PMC8197197

[ref8] Ebrahimzadeh Leylabadlo H. Ghotaslou R. Samadi Kafil H. Feizabadi M. M. Moaddab S. Y. Farajnia S.. (2020). Non-alcoholic fatty liver diseases: from role of gut microbiota to microbial-based therapies. Eur. J. Clin. Microbiol. Infect. Dis. 39, 613–627. doi: 10.1007/s10096-019-03746-1, PMID: 31828683

[ref9] Endo M. Masaki T. Seike M. Yoshimatsu H. (2007). TNF-alpha induces hepatic steatosis in mice by enhancing gene expression of sterol regulatory element binding protein-1c (SREBP-1c). Exp. Biol. Med. 232, 614–621. 17463157

[ref10] Falalyeyeva T. Chornenka N. Cherkasova L. Tsyryuk O. Molchek N. Kovalchuk O.. (2022). “Gut microbiota interactions with obesity,” in Reference Module in Food Science (Amsterdam, Netherlands: Elsevier), 201–219.

[ref11] Flores-Guerrero J. L. Groothof D. Connelly M. A. Otvos J. D. Bakker S. J. L. Dullaart R. P. F. (2019). Concentration of branched-chain amino acids is a strong risk marker for incident hypertension. Hypertension 74, 1428–1435. doi: 10.1161/hypertensionaha.119.13735 31587574

[ref12] Gao Z. Song G. Y. Ren L. P. Ma H. J. Ma B. Q. Chen S. C. (2020). Beta-catenin mediates the effect of GLP-1 receptor agonist on ameliorating hepatic steatosis induced by high fructose diet. Eur. J. Histochem. 64, 225–233. doi: 10.4081/ejh.2020.3160, PMID: 32930541 PMC7507137

[ref13] Garault P. Letort C. Juillard V. Monnet V. (2000). Branched-chain amino acid biosynthesis is essential for optimal growth of *Streptococcus thermophilus* in milk. Appl. Environ. Microbiol. 66, 5128–5133. doi: 10.1128/AEM.66.12.5128-5133.2000, PMID: 11097879 PMC92433

[ref14] Ho J. Nicolucci A. C. Virtanen H. Schick A. Meddings J. Reimer R. A.. (2019). Effect of prebiotic on microbiota, intestinal permeability, and glycemic control in children with type 1 diabetes. J. Clin. Endocrinol. Metab. 104, 4427–4440. doi: 10.1210/jc.2019-00481, PMID: 31188437

[ref15] Holecek M. (2018). Branched-chain amino acids in health and disease: metabolism, alterations in blood plasma, and as supplements. Nutr. Metab. 15:33. doi: 10.1186/s12986-018-0271-1, PMID: 29755574 PMC5934885

[ref16] Honda T. Ishigami M. Luo F. Lingyun M. Ishizu Y. Kuzuya T.. (2017). Branched-chain amino acids alleviate hepatic steatosis and liver injury in choline-deficient high-fat diet induced NASH mice. Metabolism 69, 177–187. doi: 10.1016/j.metabol.2016.12.013, PMID: 28285648

[ref17] Iwao M. Gotoh K. Arakawa M. Endo M. Honda K. Seike M.. (2020). Supplementation of branched-chain amino acids decreases fat accumulation in the liver through intestinal microbiota-mediated production of acetic acid. Sci. Rep. 10:18768. doi: 10.1038/s41598-020-75542-3, PMID: 33127939 PMC7603487

[ref18] Iwasa M. Ishihara T. Mifuji-Moroka R. Fujita N. Kobayashi Y. Hasegawa H.. (2015). Elevation of branched-chain amino acid levels in diabetes and NAFL and changes with antidiabetic drug treatment. Obes. Res. Clin. Pract. 9, 293–297. doi: 10.1016/j.orcp.2015.01.003, PMID: 25649191

[ref19] Jennison E. Byrne C. D. (2021). The role of the gut microbiome and diet in the pathogenesis of non-alcoholic fatty liver disease. Clin. Mol. Hepatol. 27, 22–43. doi: 10.3350/cmh.2020.0129, PMID: 33291863 PMC7820212

[ref20] Kaakoush N. O. Castano-Rodriguez N. Mitchell H. M. Man S. M. (2015). Global epidemiology of campylobacter infection. Clin. Microbiol. Rev. 28, 687–720. doi: 10.1128/CMR.00006-15, PMID: 26062576 PMC4462680

[ref21] Kim G. L. Lee S. Luong T. T. Nguyen C. T. Park S. S. Pyo S.. (2017). Effect of decreased BCAA synthesis through disruption of ilvC gene on the virulence of *Streptococcus pneumoniae*. Arch. Pharm. Res. 40, 921–932. doi: 10.1007/s12272-017-0931-0, PMID: 28735462

[ref22] Kong C. Gao R. Yan X. Huang L. Qin H. (2019). Probiotics improve gut microbiota dysbiosis in obese mice fed a high-fat or high-sucrose diet. Nutrition 60, 175–184. doi: 10.1016/j.nut.2018.10.002 30611080

[ref23] Le Roy T. Llopis M. Lepage P. Bruneau A. Rabot S. Bevilacqua C.. (2013). Intestinal microbiota determines development of non-alcoholic fatty liver disease in mice. Gut 62, 1787–1794. doi: 10.1136/gutjnl-2012-303816, PMID: 23197411

[ref24] Li Q. Dong K. Xu L. Jia X. Wu J. Sun W.. (2018). The distribution of three candidate cold-resistant SNPs in six minorities in North China. BMC Genomics 19:134. doi: 10.1186/s12864-018-4524-1, PMID: 29433421 PMC5809914

[ref25] Li D. J. Liu J. Hua X. Fu H. Huang F. Fei Y. B.. (2018). Nicotinic acetylcholine receptor alpha7 subunit improves energy homeostasis and inhibits inflammation in nonalcoholic fatty liver disease. Metabolism 79, 52–63. doi: 10.1016/j.metabol.2017.11.002, PMID: 29129819

[ref26] Liu C. Z. Chen W. Wang M. X. Wang Y. Chen L. Q. Zhao F.. (2020). Dendrobium officinale Kimura et Migo and American ginseng mixture: a Chinese herbal formulation for gut microbiota modulation. Chin. J. Nat. Med. 18, 446–459. doi: 10.1016/S1875-5364(20)30052-2, PMID: 32503736

[ref27] Ludwig J. Viggiano T. R. McGill D. B. Oh B. J. (1980). Nonalcoholic steatohepatitis: Mayo Clinic experiences with a hitherto unnamed disease. Mayo Clin. Proc. 55, 434–438. 7382552

[ref28] Ma J. Zhou Q. Li H. (2017). Gut microbiota and nonalcoholic fatty liver disease: insights on mechanisms and therapy. Nutrients 9:1124. doi: 10.3390/nu9101124, PMID: 29035308 PMC5691740

[ref29] Nie C. He T. Zhang W. Zhang G. Ma X. (2018). Branched chain amino acids: beyond nutrition metabolism. Int. J. Mol. Sci. 19:954. doi: 10.3390/ijms19040954, PMID: 29570613 PMC5979320

[ref30] Nyangale E. P. Mottram D. S. Gibson G. R. (2012). Gut microbial activity, implications for health and disease: the potential role of metabolite analysis. J. Proteome Res. 11, 5573–5585. doi: 10.1021/pr300637d, PMID: 23116228

[ref31] Pedersen H. K. Gudmundsdottir V. Nielsen H. B. Hyotylainen T. Nielsen T. Jensen B. A.. (2016). Human gut microbes impact host serum metabolome and insulin sensitivity. Nature 535, 376–381. doi: 10.1038/nature18646, PMID: 27409811

[ref32] Schlaepfer I. R. Joshi M. (2020). CPT1A-mediated fat oxidation, mechanisms, and therapeutic potential. Endocrinology 161:bqz046. doi: 10.1210/endocr/bqz046, PMID: 31900483

[ref33] Schoeler M. Caesar R. (2019). Dietary lipids, gut microbiota and lipid metabolism. Rev. Endocr. Metab. Disord. 20, 461–472. doi: 10.1007/s11154-019-09512-0, PMID: 31707624 PMC6938793

[ref34] Schreiber R. Xie H. Schweiger M. (2019). Of mice and men: the physiological role of adipose triglyceride lipase (ATGL). Biochim. Biophys. Acta Mol. Cell Biol. Lipids 1864, 880–899. doi: 10.1016/j.bbalip.2018.10.008, PMID: 30367950 PMC6439276

[ref35] Softic S. Meyer J. G. Wang G. X. Gupta M. K. Batista T. M. Lauritzen H.. (2019). Dietary sugars alter hepatic fatty acid oxidation *via* transcriptional and post-translational modifications of mitochondrial proteins. Cell Metab. 30:e734, 735–753.e4. doi: 10.1016/j.cmet.2019.09.003, PMID: 31577934 PMC7816129

[ref36] Strable M. S. Ntambi J. M. (2010). Genetic control of *de novo* lipogenesis: role in diet-induced obesity. Crit. Rev. Biochem. Mol. Biol. 45, 199–214. doi: 10.3109/10409231003667500, PMID: 20218765 PMC2874080

[ref37] Sun H. Wang Y. (2016). Branched chain amino acid metabolic reprogramming in heart failure. Biochim. Biophys. Acta 1862, 2270–2275. doi: 10.1016/j.bbadis.2016.09.009, PMID: 27639835

[ref38] Tokuhara D. (2021). Role of the gut microbiota in regulating non-alcoholic fatty liver disease in children and adolescents. Front. Nutr. 8:700058. doi: 10.3389/fnut.2021.700058, PMID: 34250000 PMC8267179

[ref39] van den Berg E. H. Flores-Guerrero J. L. Gruppen E. G. de Borst M. H. Wolak-Dinsmore J. Connelly M. A.. (2019). Non-alcoholic fatty liver disease and risk of incident type 2 diabetes: role of circulating branched-chain amino acids. Nutrients 11:705. doi: 10.3390/nu11030705, PMID: 30917546 PMC6471562

[ref40] Wang K. Liao M. Zhou N. Bao L. Ma K. Zheng Z.. (2019). *Parabacteroides distasonis* alleviates obesity and metabolic dysfunctions *via* production of succinate and secondary bile acids. Cell Rep. 26:e225, 222–235.e5. doi: 10.1016/j.celrep.2018.12.028, PMID: 30605678

[ref41] Yang R. Dong J. Guo H. Li H. Wang S. Zhao H.. (2013). Rapid and precise measurement of serum branched-chain and aromatic amino acids by isotope dilution liquid chromatography tandem mass spectrometry. PLoS One 8:e81144. doi: 10.1371/journal.pone.0081144, PMID: 24339906 PMC3855216

[ref42] Yang Z. Huang S. Zou D. Dong D. He X. Liu N.. (2016). Metabolic shifts and structural changes in the gut microbiota upon branched-chain amino acid supplementation in middle-aged mice. Amino Acids 48, 2731–2745. doi: 10.1007/s00726-016-2308-y, PMID: 27539648

[ref43] Yin X. Peng J. Zhao L. Yu Y. Zhang X. Liu P.. (2013). Structural changes of gut microbiota in a rat non-alcoholic fatty liver disease model treated with a Chinese herbal formula. Syst. Appl. Microbiol. 36, 188–196. doi: 10.1016/j.syapm.2012.12.009, PMID: 23453736

[ref44] Yoon M. S. (2016). The emerging role of branched-chain amino acids in insulin resistance and metabolism. Nutrients 8:405. doi: 10.3390/nu8070405, PMID: 27376324 PMC4963881

[ref45] Zeng S. L. Li S. Z. Xiao P. T. Cai Y. Y. Chu C. Chen B. Z.. (2020). Citrus polymethoxyflavones attenuate metabolic syndrome by regulating gut microbiome and amino acid metabolism. Sci. Adv. 6:eaax6208. doi: 10.1126/sciadv.aax6208, PMID: 31922003 PMC6941918

[ref46] Zhang F. Zhao S. Yan W. Xia Y. Chen X. Wang W.. (2016). Branched chain amino acids cause liver injury in obese/diabetic mice by promoting adipocyte lipolysis and inhibiting hepatic autophagy. EBioMedicine 13, 157–167. doi: 10.1016/j.ebiom.2016.10.013, PMID: 27843095 PMC5264279

[ref47] Zhao H. Zhang F. Sun D. Wang X. Zhang X. Zhang J.. (2020). Branched-chain amino acids exacerbate obesity-related hepatic glucose and lipid metabolic disorders via attenuating Akt2 signaling. Diabetes 69, 1164–1177. doi: 10.2337/db19-0920, PMID: 32184272

[ref48] Zhou M. Lu G. Gao C. Wang Y. Sun H. (2012). Tissue-specific and nutrient regulation of the branched-chain alpha-keto acid dehydrogenase phosphatase, protein phosphatase 2Cm (PP2Cm). J. Biol. Chem. 287, 23397–23406. doi: 10.1074/jbc.M112.351031, PMID: 22589535 PMC3390616

[ref49] Zhou M. Shao J. Wu C. Y. Shu L. Dong W. Liu Y.. (2019). Targeting BCAA catabolism to treat obesity-associated insulin resistance. Diabetes 68, 1730–1746. doi: 10.2337/db18-0927, PMID: 31167878 PMC6702639

